# The clinical significance of preoperative serum triglyceride, high-density lipoprotein, and low-density lipoprotein levels in lung squamous cell carcinoma

**DOI:** 10.1038/s41598-022-18589-8

**Published:** 2022-10-07

**Authors:** Zhupeng Li, Jianfeng Xu, Weizhong Feng, Zhifeng Ma, Yuanling Wu, Ting Zhu, Peng Xu, Lingjun Dong, JianYi Ding, Junqing Zhou, Guangmao Yu

**Affiliations:** Department of Cardiothoracic Surgery, Shaoxing People’s Hospital, Shaoxing Hospital, Zhejiang University, Shaoxing, 312000 Zhejiang China

**Keywords:** Cancer, Diseases, Oncology, Risk factors

## Abstract

To evaluate the prognostic role of the preoperative plasma lipid profile, including triglycerides (TG), total cholesterol (TC), low-density lipoprotein cholesterol (LDL-C), and high-density lipoprotein cholesterol (HDL-C) in patients with lung squamous cell carcinoma (LUSC) who underwent complete resection. Clinical data, including preoperative plasma profile levels, were retrospectively collected and reviewed in 300 patients with LUSC who underwent radical lung resection between 2016 and 2017. The overall survival (OS) and disease-free survival (DFS) were assessed by the Kaplan–Meier method and the Cox proportional hazards regression model. TG ≤ 1.35, HDL-C ≤ 1.17, and LDL-C ≤ 2.32 were deemed as independent preoperative risk factors for OS, and HDL-C ≤ 1.17 was an independent preoperative risk factor for DFS. In the multivariate analyses involving OS and DFS, a decreased HDL-C level was significantly associated with worse OS (HR, 0.546; 95% CI, 0.380–0.784, P = 0.001) and DFS (HR, 0.644; 95% CI, 0.422–0.981, P = 0.041). Additionally, an increased TG (HR, 0.546; 95% CI, 0.366–0.814, P = 0.003) or LDL-C (HR, 0.652; 95% CI, 0.456–0.933, P = 0.019) level was significantly associated with better OS. In patients with LUSC, decreased levels of HDL-C may predict worse outcomes for both DFS and OS, while increased TG or LDL-C levels may predict better OS.

## Introduction

Non-small cell lung cancer (NSCLC), which accounts for a majority of cases of lung cancer, is a primary subtype responsible for a predominant proportion of cancer-related deaths worldwide with an increasing incidence rate^1^. As a histology of NSCLC, lung squamous cell carcinoma (LUSC) accounts for 20–30% of all lung cancers^2^. While a high rate of recurrence after complete resection is a significant prognostic factor, the risk of metastasis and recurrence after surgery for patients with LUSC is high. To date, a variety of biomarkers have been shown to predict clinical outcomes in patients with LUSC^3,4^. For example, Masuda et al. found that laminin-5γ2 chain expression is associated with tumor cell invasiveness and patient prognosis of LUSC^5^ and Okabe et al. suggested that family with sequence similarity 83, member A (FAM83B) is a novel biomarker for the diagnosis and prognosis in LUSC^6^. Due to complicated detection equipment and expensive costs, the development of accurate, fast, and convenient predictive biomarkers for the identification of patients with a high risk of metastasis and recurrence is emerging as a significant area of investigation and may present an opportunity for improving clinical prognosis and postoperative quality of life.

Patients with cancer often present with an altered serum lipid profile. Findings from previous studies have demonstrated that a high level of serum triglycerides (TG) increases the risk of lung cancer^7,8^. Chen et al. reported a possible association between serum total cholesterol (TC) and increased risk of pancreatic carcinoma^9^, and Kitahara et al. showed a lower risk of lung cancer in males with higher serum TC than in the general population^10^. Additionally, studies have associated low high-density lipoprotein cholesterol (HDL-C) levels with an increased risk of postmenopausal breast cancer^11,12^, and the similar result has been described in patients with lung cancer^13^. A prospective cohort study of Chinese men showed that low levels of low-density lipoprotein cholesterol (LDL-C) is implicated in an increased risk of lung cancer^8^. Moreover, several recent studies have confirmed that dyslipidemia is associated with an increased risk of tumor recurrence and reduced survival for several types of cancer^9,14^. Such expositions are unsatisfactory because they do not fully explain the role of lipid metabolism in cancer development, with little evidence about the impact of lipid profiles on clinical outcomes in patients with LUSC.

Our study attempted to show the predictive role of the lipid profile on overall survival (OS) and disease-free survival (DFS) in patients with LUSC who underwent complete lung resections.

## Methods

### Patient selection

We collected and reviewed clinical data including the preoperative plasma profile levels of 300 patients with LUSC who underwent complete lung resections at Shaoxing People’s Hospital from 2016 through 2017. The study protocol was approved by the Ethics Committee of Human Experimentation in China, and all methods were performed in accordance with the relevant guidelines and regulations. All patients provided written informed consent before participation. All patients met the following eligibility criteria: (1) diagnosis of pathologically confirmed LUSC; (2) no history of cancer; (3) no treatment (including anti-cancer treatment or any other treatment that may affect blood lipids) before the serum had been obtained; and (4) collection of serum samples completed before surgery. All pathological diagnoses were independently confirmed by experienced pathologists at Shaoxing People’s Hospital.

### Patient follow-up

All patients were followed up in the clinic, and 10 patients were lost to follow up before the study was discontinued. Physical examination and laboratory blood draws were conducted every 3–6 months in the first 2 years and every 6–12 months from the third to the fifth year. The main outcome of this study was OS, defined as the time from curative resection to death. The secondary outcome was DFS, calculated from the date of radical surgery to the date of disease recurrence or diagnosis of distant metastasis^14^.

### Statistical analysis

Continuous variables are shown as means ± standard deviation (SD). The optimal cut-off values for the lipid profile determined by statistical analysis of the receiver operating characteristic (ROC) curve were used to dichotomize lipids for chi-square test and cox proportional analyses^14^. The Kaplan–Meier method and log-rank test were used to calculate the survival curve. The risk factors for survival were identified using the Cox proportional hazards model. The association between serum lipid levels and clinicopathological parameters (age, gender, smoking State, drinking state, T stage, lymph node metastasis, TNM stage, recurrence, and death) was tested with the chi-square test. In all statistical methods, *P* < 0.05 was considered significant. Data analyses were performed on SPSS 20.

### Patient study

The study has been approved by the academic ethics committee of Shaoxing People's Hospital and patients gave their informed consent before material was obtained for use in the study.

## Results

### Patient characteristics

After the eligibility review, 300 patients with LUSC who had undergone radical surgical resection were enrolled in the analysis, with their characteristics being presented in Table 1. The median follow-up time was 39 months (range: 1–70 months). The median age at resection was 60 years (range: 40–81 years), and 284 (94.7%) of the patients were male and 16 (5.3%) were female. Cancer staging was conducted according to the tumor-node-metastasis (TNM) classification for LUSC. A total of 212 patients (70.7%) were diagnosed with stage I–II disease and 88 (29.3%) were diagnosed with stage III–IV disease. A total of 138 patients (46%) died during follow-up and 109 patients (36.3%) had disease recurrence or distant metastasis.

**Table 1:** Characteristics of patients.

Characteristics	Patients	%
**Age**		
≥ 60	167	55.7
< 60	133	44.3
**Gender**		
Female	16	5.3
Male	284	94.7
**Smoking state**		
Yes	259	86.3
No	41	13.7
**Drinking state**		
Yes	177	59
No	123	41
**T stage**		
I–II	212	70.7
III–IV	88	29.3
**Lymph node metastasis**		
Yes	159	53
No	141	47
**TNM stage**		
I–II	203	67.7
III–IV	97	32.3
**Recurrence**		
Yes	109	36.3
No	181	60.3
**Death**		
Yes	138	46
No	162	54
**TG (mmol/L)**		
> 1.35	106	35.3
≤ 1.35	194	64.7
**TC (mmol/L)**		
> 3.93	165	55
≤ 3.93	135	45
**HDL-C (mmol/L)**		
> 1.17	123	41
≤ 1.17	177	59
**LDL-C (mmol/L)**		
> 2.32	200	66.7
≤ 2.32	100	33.3

### Preoperative plasma profile levels in 300 patients with LUSC

The relationship between preoperative plasma lipid profile levels and clinical parameters was analyzed (Table 2). TG level was identified to be up-regulated in patients with a higher T stage (*P* = 0.001) or TNM stage (*P* = 0.015) compared to those with a lower T stage or TNM stage. A higher HDL-C level was closely correlated with females (*P* = 0.008) and no smoking stage (*P* = 0.002). In addition, TG (*P* = 0.004) and TC (*P* = 0.047) levels were lower in patients with death status. Other clinical parameters were not significantly associated with preoperative plasma lipid profile levels, such as drinking state, lymph node metastasis, and recurrence (p > 0.05).

**Table 2:** Clinical characteristics of 300 patients with squamous cell carcinoma of lung based on TG/TC/HDL-C/LDL-C expression status.

Characteristics	TG		TC		HDL-C		LDL-C		Non-HDL-C	
	Mean ± SD	p-value	Mean ± SD	p-value	Mean ± SD	p-value	Mean ± SD	p-value	Mean ± SD	p-value
**Age**										
≥ 60	1.288 ± 0.683	0.307	4.172 ± 1.087	0.937	1.161 ± 0.303	0.513	2.620 ± 0.673	0.182	3.011 ± 1.015	0.897
< 60	1.367 ± 0.632		4.163 ± 0.899		1.138 ± 0.319		2.731 ± 0.762		3.025 ± 0.866	
**Gender**										
Female	1.269 ± 0.592	0.735	4.328 ± 1.284	0.515	1.351 ± 0.401	0.008	2.588 ± 0.834	0.639	2.977 ± 1.114	0.862
Male	1.326 ± 0.666		4.159 ± 0.991		1.140 ± 0.301		2.674 ± 0.709		3.019 ± 0.943	
**Smoking state**										
Yes	1.332 ± 0.659	0.567	4.165 ± 1.003	0.905	1.129 ± 0.296	0.002	2.680 ± 0.714	0.521	3.036 ± 0.957	0.388
No	1.268 ± 0.680		4.185 ± 1.038		1.288 ± 0.362		2.602 ± 0.727		2.898 ± 0.910	
**Drinking state**										
Yes	1.360 ± 0.667	0.253	4.225 ± 1.082	0.237	1.160 ± 0.319	0.546	2.693 ± 0.743	0.482	3.065 ± 1.039	0.292
No	1.271 ± 0.652		4.085 ± 0.885		1.138 ± 0.297		2.634 ± 0.673		2.948 ± 0.805	
**T stage**										
I–II	1.391 ± 0.728	0.001	4.195 ± 1.070	0.470	1.141 ± 0.297	0.374	2.675 ± 0.731	0.838	3.054 ± 1.019	0.291
III–IV	1.161 ± 0.422		4.103 ± 0.835		1.176 ± 0.339		2.656 ± 0.677		2.927 ± 0.760	
**Lymph node metastasis**										
Yes	1.300 ± 0.674	0.519	4.119 ± 0.936	0.372	1.140 ± 0.322	0.515	2.671 ± 0.713	0.941	2.979 ± 0.883	0.464
No	1.350 ± 0.648		4.223 ± 1.081		1.163 ± 0.295		2.657 ± 0.766		3.060 ± 1.023	
**TNM stage**										
I–II	1.381 ± 0.713	0.015	4.235 ± 1.111	0.053	1.159 ± 0.302	0.490	2.687 ± 0.750	0.517	3.076 ± 1.048	0.075
III–IV	1.203 ± 0.521		4.027 ± 0.728		1.133 ± 0.326		2.633 ± 0.636		2.894 ± 0.694	
**Recurrence**										
Yes	1.321 ± 0.703	0.875	4.202 ± 1.176	0.667	1.133 ± 0.284	0.435	2.657 ± 0.744	0.850	3.069 ± 1.146	0.485
No	1.334 ± 0.647		4.149 ± 0.878		1.161 ± 0.311		2.673 ± 0.696		2.988 ± 0.815	
**Death**										
Yes	1.206 ± 0.550	0.004	4.043 ± 1.003	0.047	1.123 ± 0.308	0.435	2.607 ± 0.725	0.166	2.920 ± 0.935	0.103
No	1.423 ± 0.730		4.274 ± 1.000		1.175 ± 0.311		2.722 ± 0.704		3.100 ± 0.959	

The Survival Rate of Patients with Increased TG or TC Levels Is Significantly Higher Than That of Patients with Decreased Levels of TG or TC.

**Figure 1:**
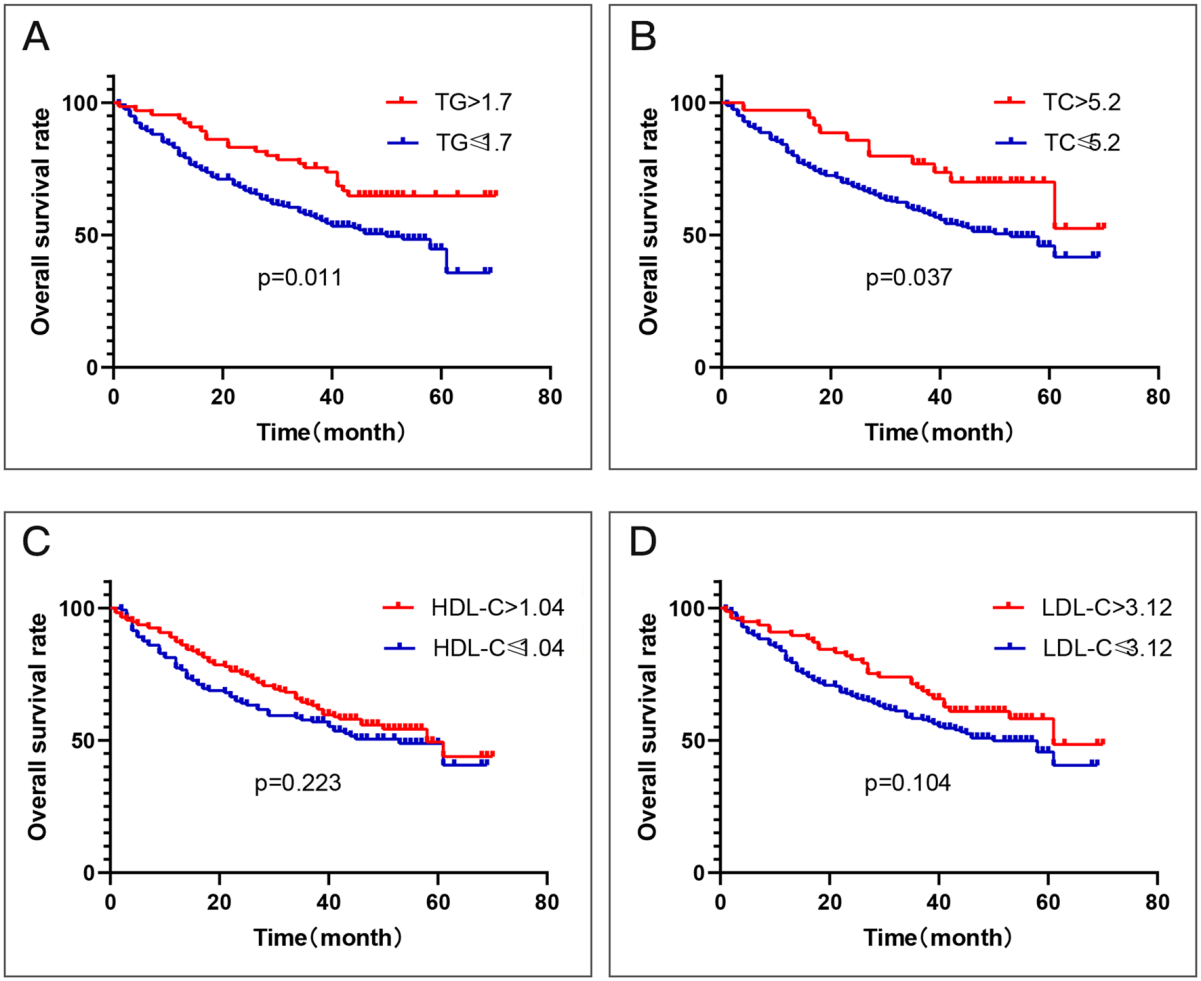
Kaplan–Meier curve for overall survival regarding low vs high TG levels (P = 0.006) (**A**); low vs high TC levels (P = 0.008) (**B**); low vs high HDL-C levels (P = 0.169) (**C**); low vs high LDL-C levels (P = 0.177) (**D**).

The Kaplan–Meier analysis results are depicted in Fig. 1. The patients with TG, TC, HDL-C, or LDL-C higher than the median were assigned to the high expression group; the other patients were assigned to the lower expression group. The OS rate was significantly higher in the TG (p = 0.006, cut-off value = 1.14) or TC (p = 0.008, cut-off value = 4.01) high expression group. In addition, the OS rate was also higher in the HDL-C or LDL-C high expression group although the difference was not significant (p > 0.05).

**Figure 2:**
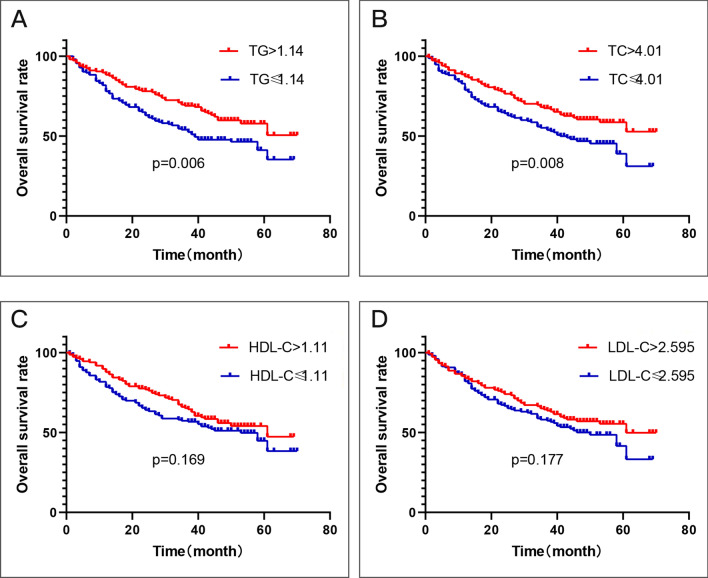
Kaplan–Meier curve for overall survival regarding normal vs disorder TG levels (P = 0.011) (**A**); normal vs disorder TC levels (P = 0.037) (**B**); normal vs disorder HDL-C levels (P = 0.223) (**C**); low vs high LDL-C levels (P = 0.104) (**D**).

We then defined patients with LUSC with TG higher than 1.7 mmol/l, TC higher than 5.2 mmol/l, HDL-C higher than 1.04 mmol/l or LDL-C higher than 3.12 mmol/l as lipid disorder group, and others as normal group. The Kaplan–Meier analysis results are plotted in Fig. 2. The OS rate was significantly higher in the TG (p = 0.011, cut-off value = 1.7) or TC (p = 0.037, cut-off value = 5.2) disorder group. In addition, the OS rate was also higher in the HDL-C or LDL-C disorder group although the difference was not significant (p > 0.05).

To investigate the potential of preoperative blood lipid level as a prognostic marker, survival Receiver operating curve (ROC) method was introduced to determine the optimal cutoff value.

### Optimal cut-off determination of lipid metabolism

ROC analysis presented that the optimal cutoff value for TG was 1.35 mmol/L (AUC 0.588, 95% CI 0.530–0.644), 3.93 mmol/L for TC (AUC 0.578, 95% CI 0.520–0.634), 1.17 mmol/L for HDL (AUC 0.551, 95% CI 0.493–0.609), and 2.32 mmol/L for LDL (AUC 0.547, 95% CI 0.489–0.605).

### The association of TG, HDL-C, and LDL-C levels with clinical characteristics

The relationships between the clinicopathological features of LUSC and TG, HDL-C, and LDL-C levels are summarized in Tables 3, 4 and 5. The Chi-square test showed the TG level was significantly related to the tumor (T) stage (*P* = 0.003), TNM stage (*P* = 0.008), and death (*P* = 0.002) (Table 3). However, no significant associations were reported between TG levels with other clinicopathological parameters, including smoking status, lymph node metastasis, and tumor recurrence (all *P* > 0.05; Table 3). The HDL-C level was significantly associated with sex (*P* = 0.020), smoking status (*P* = 0.002), tumor recurrence (*P* = 0.031), and death (*P* = 0.013) (Table 4). The LDL-C level was significantly associated with death (*P* = 0.014; Table 5).

**Table 3:** Relationship between TG concentration and clinical characteristics in 300 patients with squamous cell carcinoma of lung based on chi-square test.

Characteristics	TG > 1.35	TG ≤ 1.35	P
**Age**			0.144
≥ 60	53	114	
< 60	53	80	
**Gender**			0.852
Female	6	10	
Male	100	184	
**Smoking state**			0.382
Yes	94	165	
No	12	29	
**Drinking state**			0.273
Yes	67	110	
No	39	84	
**T stage**			0.003
I–II	86	126	
III–IV	20	68	
**Lymph node metastasis**			0.598
Yes	54	105	
No	52	89	
TNM stage			0.008
I–II	82	121	
III–IV	24	73	
**Recurrence**			0.148
Yes	33	76	
No	70	111	
**Death**			0.002
Yes	36	102	
No	70	92

**Table 4:** Relationship between HDL-C concentration and clinical characteristics in 300 Patients With squamous cell carcinoma of lung based on chi-square test.

Characteristics	HDL-C > 1.17	HDL-C ≤ 1.17	P
**Age**			0.900
≥ 60	69	98	
< 60	54	79	
**Gender**			0.020
Female	11	5	
Male	112	172	
**Smoking state**			0.002
Yes	97	162	
No	26	15	
**Drinking state**			0.918
Yes	73	104	
No	50	73	
**T stage**			0.592
I–II	89	123	
III–IV	34	54	
**Lymph node metastasis**			0.606
Yes	63	96	
No	60	81	
**TNM stage**			0.089
I–II	90	113	
III–IV	33	64	
**Recurrence**			0.031
Yes	36	73	
No	83	98	
**Death**			0.013
Yes	46	92	
No	77	85

**Table 5:** Relationship between LDL-C concentration and clinical characteristics in 300 Patients With squamous cell carcinoma of lung based on chi-square test.

Characteristics	LDL-C > 2.32	LDL-C ≤ 2.32	P
**Age**			0.742
≥ 60	110	57	
< 60	90	43	
**Gender**			0.467
Female	12	4	
Male	188	96	
**Smoking state**			0.905
Yes	173	86	
No	27	14	
**Drinking state**			0.213
Yes	123	54	
No	77	46	
**T stage**			0.720
I–II	140	72	
III–IV	60	28	
**Lymph node metastasis**			0.624
Yes	108	51	
No	92	49	
**TNM stage**			0.541
I–II	133	70	
III–IV	67	30	
**Recurrence**			0.621
Yes	71	38	
No	123	58	
**Death**			0.014
Yes	82	56	
No	118	44	

### The prognostic significance of clinical characteristics in LUSC

In the univariate analyses, a significant correlation between T stage, lymph node metastasis, TNM stage, levels of TG, TC, HDL-C, and LDL-C, and OS were detected. Additionally, we found a correlation between T stage, TNM stage, HDL-C levels, and DFS. In multivariate analysis, we observed significant associations of T stage, LNM, and levels of TG, HDL-C, and LDL-C with OS (Table 6). In addition, associations between T stage and HDL-C levels with DFS were observed (Table 7). The multivariate analysis was conducted based on age at resection, sex, smoking status, drinking status, T stage, lymph node metastasis, TNM stage, and levels of TG, TC, HDL-C, and LDL-C.

**Table 6:** Univariate and multivariate cox proportional analysis with overall survival.

	Univariate analysis		Multivariate analysis	
Parameter	HR (95% CI)	P	HR (95% CI)	P
**Age**	1.024(0.732–1.432)	0.890		
≥ 60				
< 60				
**Gender**	0.816(0.360–1.851)	0.627		
Female				
Male				
**Smoking state**	0.998(0.600–1.660)	0.995		
Yes				
No				
**Drinking state**	1.008(0.716–1.418)	0.964		
Yes				
No				
**T stage**	0.437 (0.311–0.615)	0.000	0.444(0.315–0.627)	0.000
I–II				
III–IV				
**Lymph node metastasis**	1.738 (1.229–2.457)	0.002	1.819(1.281–2.581)	0.001
Yes				
No				
**TNM stage**	0.404(0.288–0.566)	0.000		
I–II				
III–IV				
**TG (mmol/L)**	0.497(0.340–0.728)	0.000	0.546(0.366–0.814)	0.003
> 1.35				
≤ 1.35				
**TC (mmol/L)**	0.621(0.444–0.868)	0.005		
> 3.93				
≤ 3.93				
**HDL-C (mmol/L)**	0.639(0.449–0.911)	0.013	0.546(0.380–0.784)	0.001
> 1.17				
≤ 1.17				
**LDL-C (mmol/L)**	0.642(0.457–0.903)	0.011	0.652(0.456–0.933)	0.019
> 2.32				
≤ 2.32

**Table 7:** Univariate and multivariate cox proportional analysis with disease-free survival.

Parameter	Univariate analysis		Multivariate analysis	
	HR (95% CI)	P	HR (95% CI)	P
**Age**	1.167(0.777–1.755)	0.456		
≥ 60				
<60				
**Gender**	0.536(0.170–1.692)	0.288		
Female				
Male				
**Smoking state**	1.050(0.573–1.925)	0.874		
Yes				
No				
**Drinking state**	1.004(0.668–1.509)	0.985		
Yes				
No				
**T stage**	0.460(0.305–0.694)	0.000	0.457(0.303–0.690)	0.000
I–II				
III–IV				
**Lymph node metastasis**	1.243(0.831–1.860)	0.290		
Yes				
No				
**TNM stage**	0.518(0.344–0.779)	0.002		
I–II				
III–IV				
**TG (mmol/L)**	0.734(0.481–1.119)	0.151		
> 1.35				
≤ 1.35				
**TC (mmol/L)**	0.705(0.472–1.052)	0.087		
> 3.93				
≤ 3.93				
**HDL-C (mmol/L)**	0.650(0.426–0.990)	0.045	0.644(0.422–0.981)	0.041
> 1.17				
≤ 1.17				
**LDL-C (mmol/L)**	0.838(0.550–1.277)	0.411		
> 2.32				
≤ 2.32				

### The prognostic significance of the serum lipid profile in LUSC

**Figure 3:**
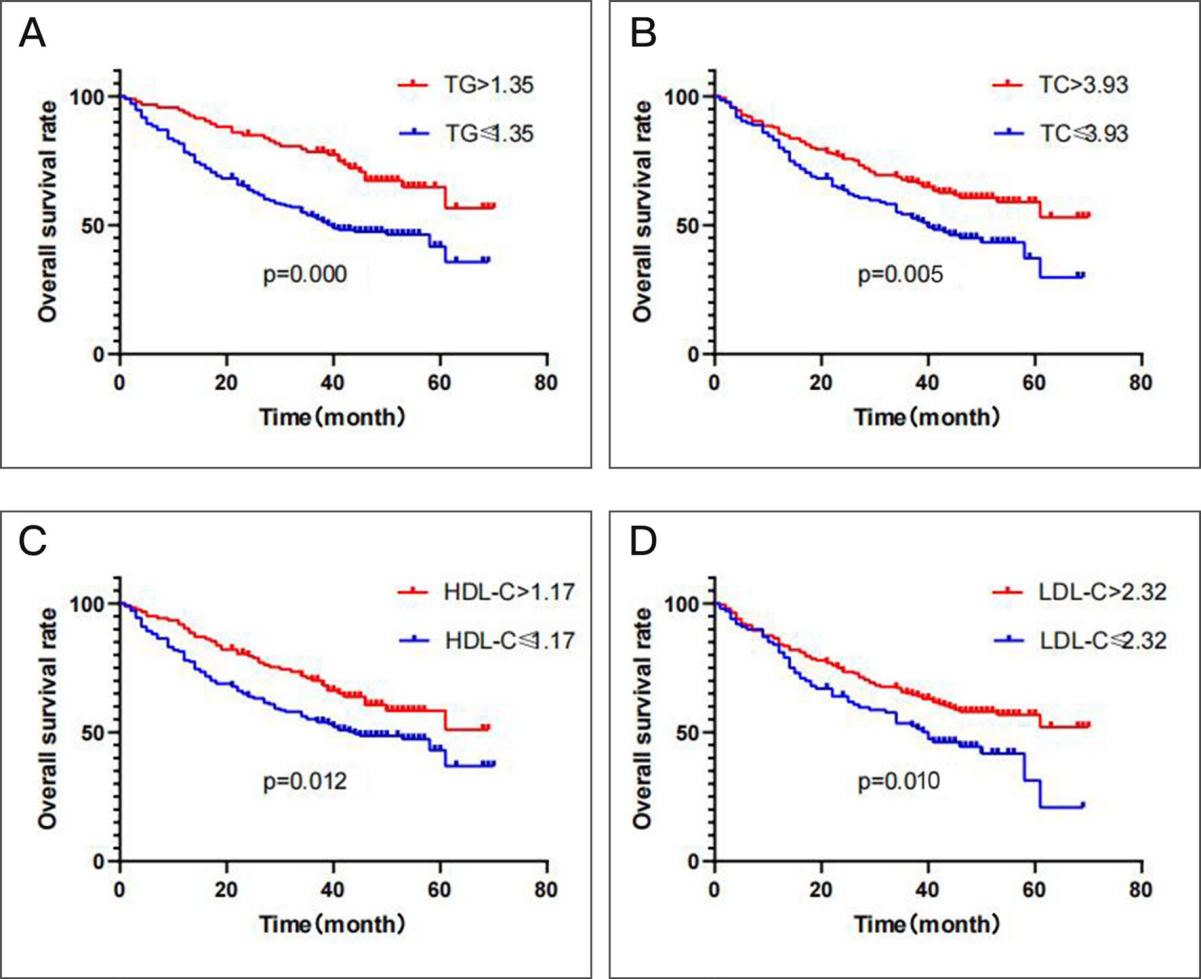
Kaplan–Meier curve for overall survival regarding low vs high TG levels (P = 0.000) (**A**); low vs high TC levels (P = 0.005) (**B**); low vs high HDL-C levels (P = 0.012) (**C**); low vs high LDL-C levels (P = 0.010) (**D**).

Among the 300 patients, 102 of 194 (52.6%) patients had a TG level ≤ 1.35 mmol/L, and 36 of 106 (34.0%) patients had a TG level > 1.35 mmol/L (P = 0.002); 74 of 135 (54.8%) patients had a TC level ≤ 3.93 mmol/L, and 64 of 165 (38.8%) patients had a TC level > 3.93 mmol/L (P = 0.006); 92 of 177 (52.0%) patients had an HDL-C level ≤ 1.17 mmol/L, and 46 of 123 (37.4%) patients had an HDL-C level > 1.17 mmol/L (P = 0.013); 56 of 100 (56.0%) patients had an LDL-C level ≤ 2.32 mmol/L, and 82 of 200 (41.0%) patients had an LDL-C level > 2.32 mmol/L (P = 0.014) who died. In the univariate Cox proportional analysis, a decreased TG (HR, 0.497; 95% CI, (0.340–0.728), P = 0.000), HDL-C (HR, 0.639; 95% CI, (0.449–0.911), P = 0.013), or LDL-C (HR, 0.642; 95% CI, (0.457–0.903), P = 0.011) level was significantly associated with decreased OS (Table 6; Fig. 3) and this finding remained significant in the multivariate analysis (HR, 0.546; 95% CI, 0.380—0.784, P = 0.001) that included T stage and lymph node metastasis. In addition, an increased TC level (HR, 0.621; 95% CI, (0.444–0.868), P = 0.005; Table 6; Fig. 3) and III-IV TNM stage (HR, 0.404; 95% CI, (0.288–0.566), P = 0.000) were statistically linked with increased OS in the univariate Cox proportional analysis. However, in the multivariate analysis, TC levels were not statistically significant including TNM stage (P > 0.05; Table 6). Other clinical parameters showed no significant difference in the results from either the univariate or multivariate analysis, such as gender and smoking state (P > 0.05; Table 6).

**Figure 4:**
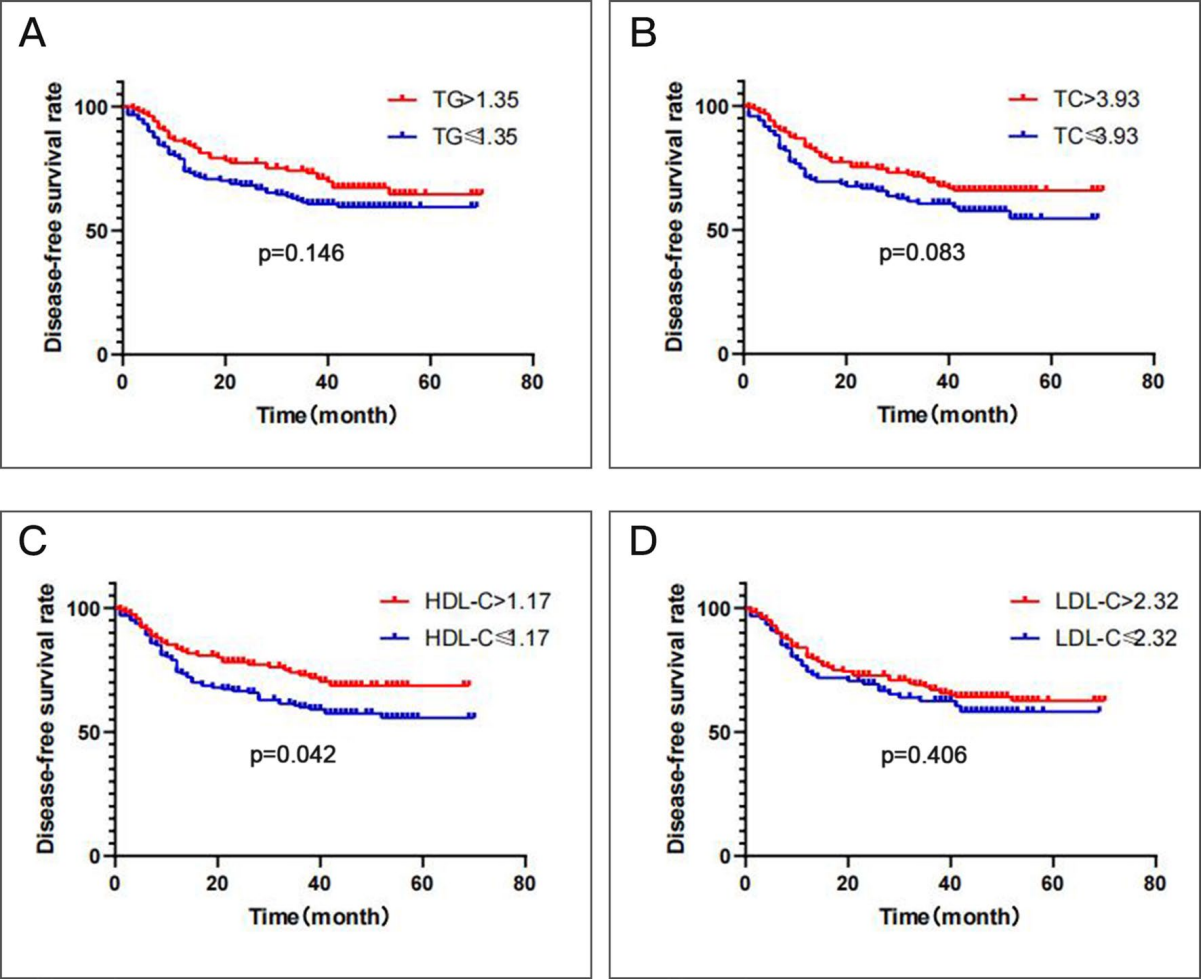
Kaplan–Meier curve for disease-free survival regarding low vs high TG levels (P = 0.146) (**A**); low vs high TC levels (P = 0.083) (**B**); low vs high HDL-C levels (P = 0.042) (**C**); low vs high LDL-C levels (P = 0.406) (**D**).

Regarding DFS, local recurrence or distant metastasis after radical surgical resection was diagnosed in 76 of 187 (40.6%) patients with a TG level ≤ 1.35 mmol/L and in 33 of 103 (32.0%) patients with a TG level > 1.35 mmol/L (*P* > 0.05); in 55 of 129 (42.6%) patients with a TC level ≤ 3.93 mmol/L and in 54 of 161 (33.5%) patients with a TC level > 3.93 mmol/L (*P* > 0.05); in 73 of 171 (42.7%) patients with an HDL-C level ≤ 1.17 mmol/L and in 36 of 119 (30.3%) patients with an HDL-C level > 1.17 mmol/L (*P* = 0.031); in 38 of 96 (39.6%) patients with an LDL-C level ≤ 2.32 mmol/L and in 71 of 194 (36.6%) patients with an LDL-C level > 2.32 mmol/L (*P* > 0.05). In the univariate Cox proportional analysis, A decreased HDL-C (HR, 0.650; 95% CI, (0.426–0.990), P = 0.045; Table 7; Fig. 4) level was significantly correlated with decreased DFS and the significance of this finding remained in the multivariate analysis (HR, 0.546; 95% CI, 0.380–0.784, P = 0.001) including the T stage. In addition, III-IV TNM stage was statistically linked with increased DFS (HR, 0.518; 95% CI, (0.344–0.779), *P* = 0.002), although there was no statistical significance of TNM stage in the multivariate analysis (P > 0.05; Table 7). TG, TC, and HDL-C showed no significant difference in the results from either the univariate or multivariate analysis (Table 7; Fig. 4) including age, gender, smoking state, and drinking State (P > 0.05; Table 7).

## Discussion

In view of the poor prognosis of patients with LUSC and the high tumor recurrence rate after resection, investigations on risk factors for tumor prognosis and recurrence are warranted, so as to support early intervention, improve monitoring, reduce tumor recurrence rate, and prolong patient survival time^15,16^. On account of the close relationship between blood lipids and tumorigenesis or tumor recurrence, we were interested to see if a relationship existed between lipid profile and the prognosis of Chinese patients with LUSC.

In our present study, the result that a lower TG level indicated a poorer prognosis in patients with LUSC and was associated with advanced cancer stages may be elucidated by the poor nutritional status and high-energy consumption of patients with advanced tumors^17,18^. The present study suggests, for the first time, an association of a low TG level with reduced survival of lung cancer.

Additionally, we manifested that lower TC levels indicated a poor OS in patients with LUSC. Cholesterol is essential for maintaining the integrity, fluidity, and function of the cell membrane, including signal transduction^19,20^. Low cholesterol has been proposed to be associated with suppressed immunity, upregulated activity or responsiveness of the mevalonate pathway and increased activity of nuclear transcription factor NF-κB, promoting the initiation and progression of cancer^8^. High cholesterol levels reduce the risk of death in patients with advanced NSCLC who have the epidermal growth factor receptor (EGFR) gene mutation^21^, which is line with our results. But the question of whether TC is an independent risk factor for the prognosis of patients with LUSC warrants further study.

There is emerging evidence that HDL-C may be linked to the tumorigenesis and progression of cancers^14,22^. The initial observations prompted us to investigate the relation between HDL-C and LUSC. We revealed that increased pre-operative plasma HDL-C levels were associated with increased DFS and OS after radical surgery. Lower HDL-C levels were related to sex and smoking status but not cancer stages. Additional support for the role of HDL-C comes from recent publications, which demonstrated that HDL-C may affect the occurrence of cancer by participating in the reverse transport of cholesterol^23^, affecting the entry into the cell cycle^24^, and regulating apoptosis and inflammatory response^25,26^. From previous studies, two possible molecular mechanisms have been suggested for the relation between HDL-C and the relapse of LUSC and poor survival. Serum HDL-cholesterol levels are negatively associated with cholesterol levels in the tumor tissues of patients, revealing the possibility that lower HDL levels are resulted from the increased cholesterol metabolism in the tissue of proliferating tumors^27^. The other is that the increase in HDL level is related to the decrease of pro-inflammatory cytokines that can promote cell proliferation and inhibit apoptosis, and also related to the increase of anti-inflammatory cytokines^28,29^.

LDL-C is another major indicator for monitoring lipoprotein cholesterol in addition to HDL-C. Our study found that low expression of LDL-C in patients with LUSC was substantially associated with poor OS. Although there are limited reports on the relationship between LDL-C and the prognosis of tumor patients, Zhou et al. observed that higher LDL level is an important independent prognostic factor for poor OS in patients with small-cell lung cancer^30^. The inconsistency of the role of LDL level may be due in part to the different pathological basis of LUSC and small cell lung cancer. Furthermore, the treatment of LUSC is mainly surgery in our study, while either radiotherapy or chemotherapy is used for treating small cell lung cancer in the study of Zhou et al.^30^.

Previous studies have demonstrated that most patients with non-small cell lung cancer suffer from malnutrition^19^. In addition, even patients with normal nutrition may suffer from malnutrition in the early postoperative period, which may lead to poor wound healing and reduced immunity after surgery, resulting in an increased risk of postoperative complications including bronchial leakage, postoperative infection, respiratory asthenia, and ultimately poor OS and progression-free survival^31,32^. Lipids, as an essential nutrient of human body, the absence of which can slow down or even stop all functions of human body. This will lead to the increased incidence rate and mortality.

Being limited to this retrospective study, blood lipids were not measured again after surgery. Another deficiency existed that some patients with hyperlipidemia received lipid-lowering treatment after surgery. At present, a much debated issue is that whether taking statins after surgery can improve the prognosis of lung cancer patients. Whilst some studies have proven that taking statins before diagnosis can reduce the specific mortality of lung cancer patients and prolong the survival time of patients^33,34^, some other scholars believe no superiority of taking statins over no-statins in lowering risk of lung cancer^35^.

## Conclusions

In summary, our research indicated that blood lipid levels were substantially correlated with LUSC generation and development and may be helpful in the identification of, and follow-up in, high-risk patients with LUSC. Therefore, TG, HDL-C, and LDL-C levels at diagnosis can be considered prognostic factors for LUSC.

## Supplementary Information

Supplementary Information.

## Data Availability

The data used to support the findings of this study is included in the article and supplementary file.
